# Interfaces between Cranial Bone and AISI 304 Steel after Long-Term Implantation: A Case Study of Cranial Screws

**DOI:** 10.1021/acsbiomaterials.4c00309

**Published:** 2024-06-20

**Authors:** Natália Luptáková, Václav Dlouhý, Dinara Sobola, Stanislava Fintová, Adam Weiser, Vladimír Beneš, Antonín Dlouhý

**Affiliations:** † Institute of Physics of Materials, AS CR, v. v. i., Žižkova 513/22, Brno 61662, Czech Republic; ‡ Department of Neurosurgery, Second Faculty of Medicine, Charles University and University Hospital Motol, V Úvalu 84, Prague 150 06, Czech Republic

**Keywords:** stainless steel AISI 304, implant, biocorrosion, peri-implant bone, remodelling

## Abstract

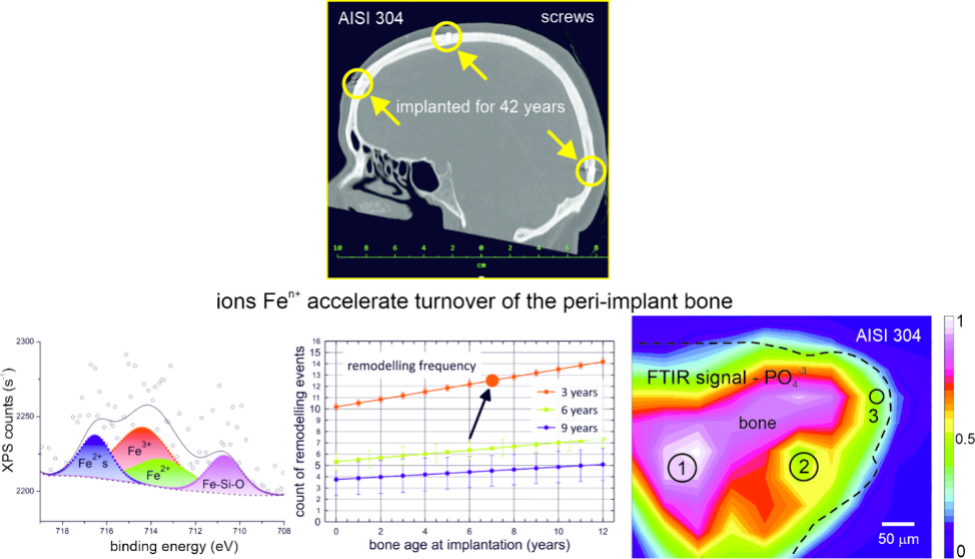

Interfaces between AISI 304 stainless steel screws and cranial bone were investigated after long-term implantation lasting for 42 years. Samples containing the interface regions were analyzed using state-of-the-art analytical techniques including secondary ion mass, Fourier-transform infrared, Raman, and X-ray photoelectron spectroscopies. Local samples for scanning transmission electron microscopy were cut from the interface regions using the focused ion beam technique. A chemical composition across the interface was recorded in length scales covering micrometric and nanometric resolutions and relevant differences were found between peri-implant and the distant cranial bone, indicating generally younger bone tissue in the peri-implant area. Furthermore, the energy dispersive spectroscopy revealed an 80 nm thick steel surface layer enriched by oxygen suggesting that the AISI 304 material undergoes a corrosion attack. The attack is associated with transport of metallic ions, namely, ferrous and ferric iron, into the bone layer adjacent to the implant. The results comply with an anticipated interplay between released iron ions and osteoclast proliferation. The interplay gives rise to an autocatalytic process in which the iron ions stimulate the osteoclast activity while a formation of fresh bone resorption sites boosts the corrosion process through interactions between acidic osteoclast extracellular compartments and the implant surface. The autocatalytic process thus may account for an accelerated turnover of the peri-implant bone.

## 1 Introduction

Interactions between bone tissue and metallic implants subjected to complex loading and biochemical conditions have been a subject of intensive research for many decades.^1,2^ From the point of view of clinical practice, the interest is mainly motivated by frequent postsurgery trauma and long-term worsening status of the implants.^3^ A metallic implant always represents an element disparate to the environment of the living bone. If not sufficiently inert, the adverse interactions between the implant and the adjacent tissue may lead to the implant rejection in the short-term.^4^ On the other hand, decades of clinical practice has shown that implants can be accepted and function in the bioenvironment for many years.^5,6^ In view of future development in the field, positive and negative implantation outcomes need to be fully understood focusing on processes that govern the interactions on micro- and nanostructural length scales.^1^

Due to a growth of life expectancy, implanted materials would be expected to withstand correspondingly longer exposures to the aggressive bioenvironments. However, data on the long-term performance of the metallic components implanted into bone tissues for time periods exceeding 30 years are rather scarce.^7^ One reason is that, in a majority of cases, an aseptic loosening of the implants is almost inevitable within 15–20 years after the surgery.^8,9^ Moreover, the corresponding studies frequently target situations in which a combination of wear and biochemical corrosion contributes to osteolysis and implant failure, like in the case of hip prostheses.^10−13^ In order to investigate mainly the long-term effects of biochemical corrosion in vivo without intervention of wear, cranial implants represent a proper research alternative. Nevertheless, concerning long-term data on cranial implants, the state of affairs is similar to the hip prostheses.^14−18^

A case study of a Sherman plate, fabricated from 304 stainless steel and extracted from a patient’s arm after being in vivo for 38 years, ruled out any apparent corrosion of the implant.^7^ However, the conclusion relied on energy dispersive spectroscopy (EDS) data acquired just from the implant surface without any attempt to characterize a chemical profile in deeper layers under the surface. Moreover, the analyses^7^ focused solely on the metallic side opposite to the forearm bones and did not address chemical changes of the bone in contact with the plate. In order to overcome these omissions, one objective of the present study is to characterize the local chemical compositions in the implant and bone layers at and next to the common interface after long-term interactions exceeding 30 years. We take advantage of an immense development of analytical tools achieved during last two decades^19−21^ which improved lateral resolution to a level not accessible to the former investigation.^7^

Concerning prospective biocorrosion, cells come into a contact with the metallic interface and become exposed to the inorganic material during bone remodelling cycles.^22^ In a distant bone (bone far from the metallic interface), a typical bone turnover period (remodelling cycle plus a quiescence period) was estimated as 25 and 4 years for cortical and trabecular bones, respectively.^23^ In contrast to the distant bone, the bone turnover at the bone–implant interfaces is less well understood.^19^ The literature data suggest that the mutual bone–implant interactions alter both parts of the system. While the metal surface is subjected to biocorrosion and possibly also wear due to chemical attack and mechanical loadings,^24,25^ a flux of metallic ions from the implant into the bone tissue may modify bone homeostasis in a complex way.^1^

In contrast to the preceding study,^7^ which only considered body fluids (pH ∼ 7.4) as a potential agent causing corrosion, we suspect that stronger acidity within osteoclast extracellular compartments (pH ∼ 5), activated during the peri-implant bone resorption, may provide much stronger effects on the implant surfaces and, in the long term, generate a higher concentration of the biocorrosion products. Understanding the long-term effects of biocorrosion products on the peri-implant bone homeostasis is thus a second objective of the present investigation. Our case study focuses on AISI 304 cranial screws which were implanted in the lamina externa for 42 years and thus were mainly exposed to biochemical attack with only negligible or no contribution due to mechanical wear. The analysis of the bone–screw interface is performed using state-of-the-art electron microscopy and spectroscopy techniques.

## 2 Experimental Section

### 2.1 Implanted–Explanted Material

**Figure 1:**
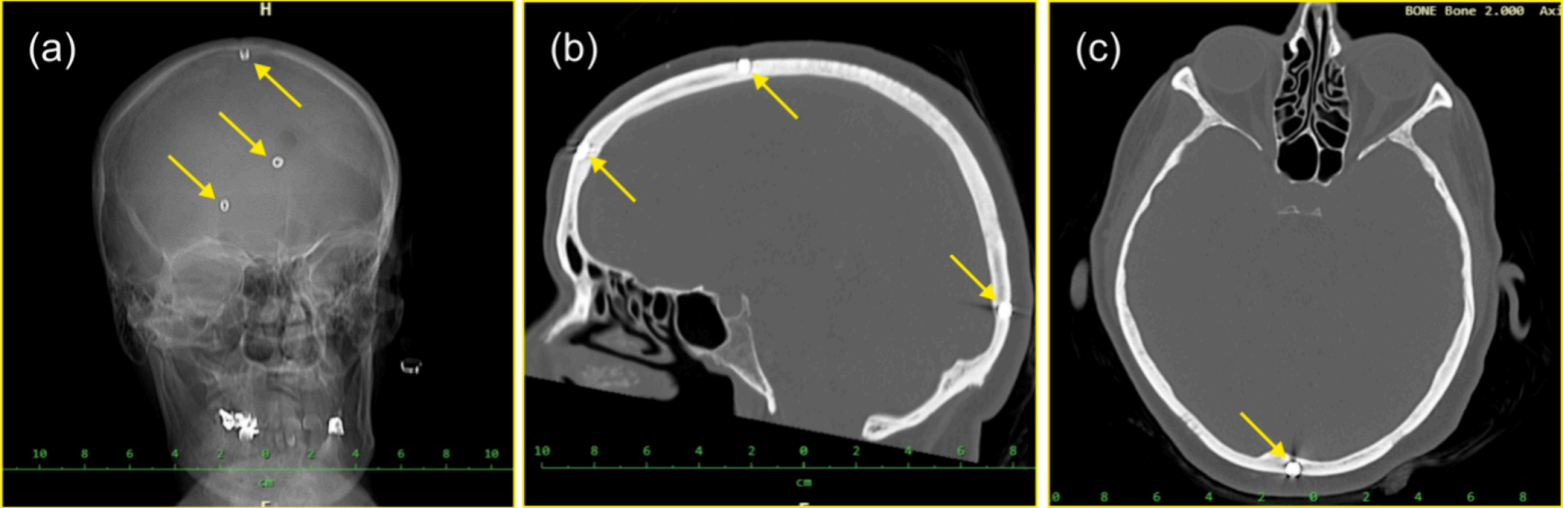
Arrows in the CT images indicate positions in which the navigation screws rested for 42 years. Two bone layers, namely, lamina externa (cortical) and diploe (cancellous) hosted the metallic material. (a) Front view. (b) Side view. (c) Top view.

Interfaces formed between the cranial bone and stainless steel AISI 304 screws, which rested in the bone for 42 years, were investigated. In 1978, the 26 year old patient underwent a special thermolesion of the thalamus. With respect to the year of the surgery and methods available at the time, cutting tools were navigated with stereotactic maps fixed to the patient’s head by means of the screws. Before the thermolesion, the screws were implanted to the frontal, parietal, and occipital bone and left in place after the operation, see Figure 1.

**Figure 2:**
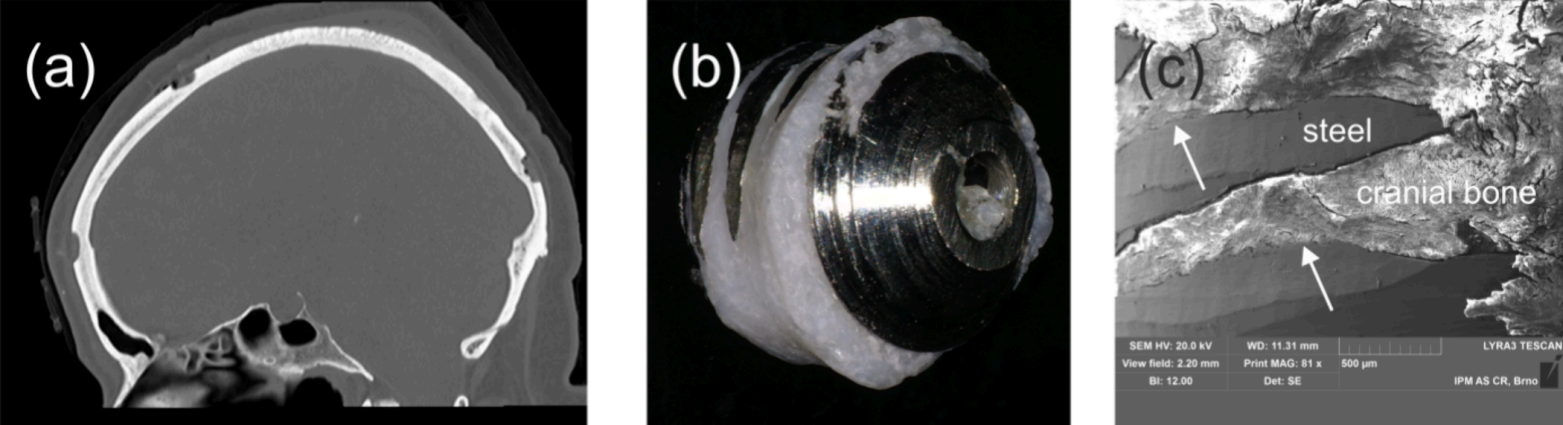
(a) Postoperation CT image documenting former locations of screws after their removal together with the surrounding bone. (b) Macroscopic view of the screw imbedded in the bone tissue after being explanted from the cranial bone. (c) Partially detached interfaces between bone and the screw, locations of a good adhesion are indicated by arrows; for further details see text.

Recently, at the age of 68, the patient exhibited an impairing neurological status and was recommended for an MRI investigation, before which the screws were explanted. During the explantation, a special small drill (B - Braun Elan 4) penetrated the lamina externa and diploe such that the screws were recovered from the former application positions. As is documented by the CT, light, and scanning electron microscopy images shown in Figure 2, pieces of the cranial bone remained distributed over the explanted screws. White arrows in Figure 2c mark interface locations with a full adhesion between the steel and bone parts. Where appropriate, analytical investigations reported in the present study focus on this type of interface segment. In view of different properties exhibited by the individual laminas and diploe,^26,27^ it is important to highlight that our focus is on the cortical bone tissue which originally belonged to the lamina externa. For this bone tissue, we compare characteristics far from the bone–implant interface (distant bone = tissue situated more than about 100 μm from the interface) and close to the interface (peri-implant bone = tissue within an about 100 μm thick layer adjacent to the implant).

### 2.2 Characterization Methods

#### 2.2.1 Metallographic Surfaces

The screws were divided in halves by an axial cut (a diamond saw Brilliant 220 from Metalco) using water as a coolant. One half of the screw was subsequently mounted into conductive resin (Struers) and flattened on emery papers with a 4000-grit finish. In the next step, the surface was mechanically polished in a vibratory polisher Saphir 330 ATM using a colloidal silica with particle size 50 nm. After the polishing, the surfaces were rinsed several times by a degreasing water solution, ultrasonically cleaned for 5 min in the acetone bath, and finally rinsed by deionized water and dried in a warm airflow in order to remove any possible contamination by the cutting and polishing media. The metallographic surfaces were then investigated by conventional light microscopy (LM), scanning electron microscopy (SEM), secondary ion mass spectroscopy (SIMS), Fourier-transform infrared (FTIR) and Raman (RS) spectroscopies, and X-ray photoelectron spectroscopy (XPS). Right after the cutting, the second half of the screw was only cleaned with deionized water without any additional polishing treatment. This part of the screw was inspected for implant–bone interface locations suitable for focused ion beam (FIB) cutting; see section 2.2.4 for details.

#### 2.2.2 SIMS

Depth profiles tracing a chemical composition along the interfaces were acquired by SIMS using a dual beam TOF-SIMS5 setup (IONTOF, Germany). The material was etched away by O_2_^+^ ions in intervals lasting for 0.13 s. The O_2_^+^ beam energy and current were, respectively, 2 keV and 600 nA and the area of the forming crater was 500 × 500 μm^2^. The etching period was then followed by a period of a chemical analysis. For the chemical analysis, a liquid Bi metal ion gun with a fine-focused primary Bi^+^ ion beam was used to knocked out secondary ions from the first two monolayers of the fresh surface in the bone and steel parts. The Bi^+^ ion beam parameters were 30 keV and 0.1–0.3 pA. Duration of the chemical analysis period was 3.6 s followed by 1 s of resting time before the next interface layer was taken away by the O_2_^+^ beam. In order to avoid effects associated with the original surface and building of crater edges, an analyzed volume of 350 × 350 × 0.75 μm^3^ was situated deep below the metallographic surface and fully inside the crater. In total, the combined action of the two beams resulted in a removal of a 15 μm thick layer of the steel part in 12000 s. SurfaceLab soft version 7.1.124633 (IONTOF, Germany) was used for processing of the chemical data.

#### 2.2.3 Vibrational Spectroscopies and XPS

The spectroscopies provided insight into chemical bonding at different bone locations. FTIR measurements were performed using a wide band Mercury Cadmium Telluride (MCT) detector (Bruker, Billerica, MA, USA) cooled by liquid nitrogen. The MCT detector together with a KBr beam splitter provided a spectral resolution of 4 cm^–1^ and a mapping lateral resolution of 30 μm. The OPUS 6.0 software from Bruker was employed for data processing. The Raman spectroscopy was carried out using the WITec confocal Raman imaging system, alpha300 (WITec, Ulm, Germany). The excitation laser operated at a wavelength of 532 nm and power of 1 mW. An integration time for one point was 10 s with a mapping lateral resolution of 1 μm. The map and spectra were processed by the Project FIVE 5.2.3.78 software (WITec, Ulm, Germany). An AXIS Supra X-ray photoelectron spectrometer (Kratos Analytical, Manchester, UK) was used with the emission current 15 mA and aperture 110 μm. The general calibration of the spectrometer was performed under ultrahigh vacuum conditions utilizing high purity standards, namely, a silver line Ag 3d_5/2_ at the energy 368.2 eV, a gold line Au 4f_7/2_ at the energy 84.0 eV, and a copper line Cu 2p_3/2_ at the energy 933.0 eV. Furthermore, during the evaluation of each spectrum, the C 1s line was first set at 284.8 eV (C–C bond) and the correctness of the calibration was then rechecked by the position of the O 1s peak at 532 eV. All these steps yielded a correct scale for a subsequent analysis of other binding energies. The data were fitted using Casa XPS software, version 2.3.17PR1.1 (Casa Software Ltd.).

#### 2.2.4 Light and Electron Microscopy

Standard light microscopy (digital microscope Olympus DSX1000), SEM, and scanning transmission electron microscopy (STEM) observations were performed. The SEM experiments ran on a dual beam LYRA 3 XMU FEG/SEM-FIB microscope from TESCAN. This microscope is furnished with a FIB facility suitable for cutting of thin lamellae by a Ga^+^ ion beam. These thin samples with an estimated volume of 10 × 10 × 0.012 μm^3^ allow direct STEM observations of the metal–bone interface with high spatial resolution.

**Figure 3:**
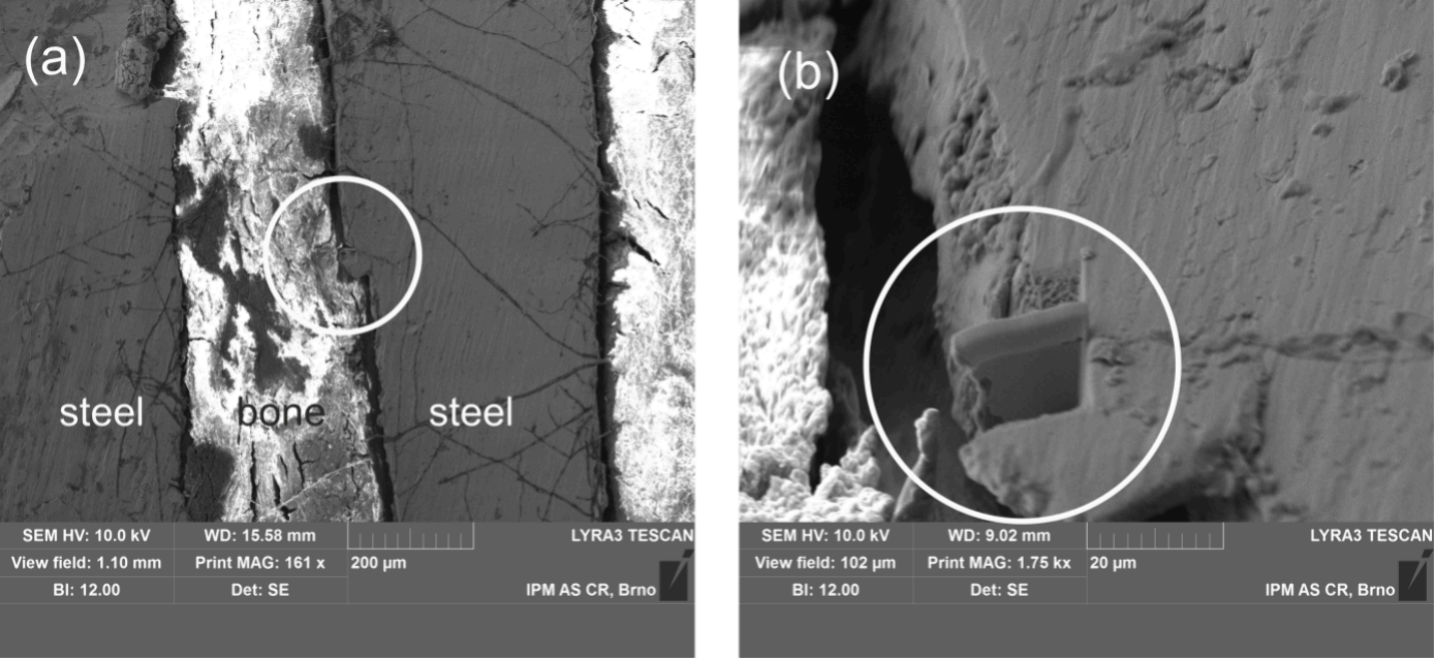
Interface between AISI 304 steel and bone. (a) The circle indicates a location from where the STEM lamella was cut by FIB. (b) Detail showing the STEM lamella before a lift-up. See text for further details.

In the present work, the plane of the FIB lamellae with estimated area of 10 × 10 μm^2^ was oriented perpendicular to the original surface of the AISI 304 screw as is indicated in Figure 3. A platinum protection layer was deposited on the surface across the transition between the screw and bone prior to the FIB machining. This protection layer prevented the surface structures from any possible damage during the FIB operations. A micromanipulator was used to lift out the lamella and weld it to a copper support which is designed for placement in STEM sample holders. Subsequent STEM investigations were performed in a JEM-2100 F microscope from JEOL operated at 200 kV with an analytical system AZtec from Oxford Instruments. In order to obtain nanometer resolved chemical data, a fine incident electron probe with a typical size of 0.5 nm was used during the EDS experiments. Quantitative assessment of the selected area diffraction (SAD) data was performed using JEMS^28^ and ACC^29^ software applications.

**Figure 4:**
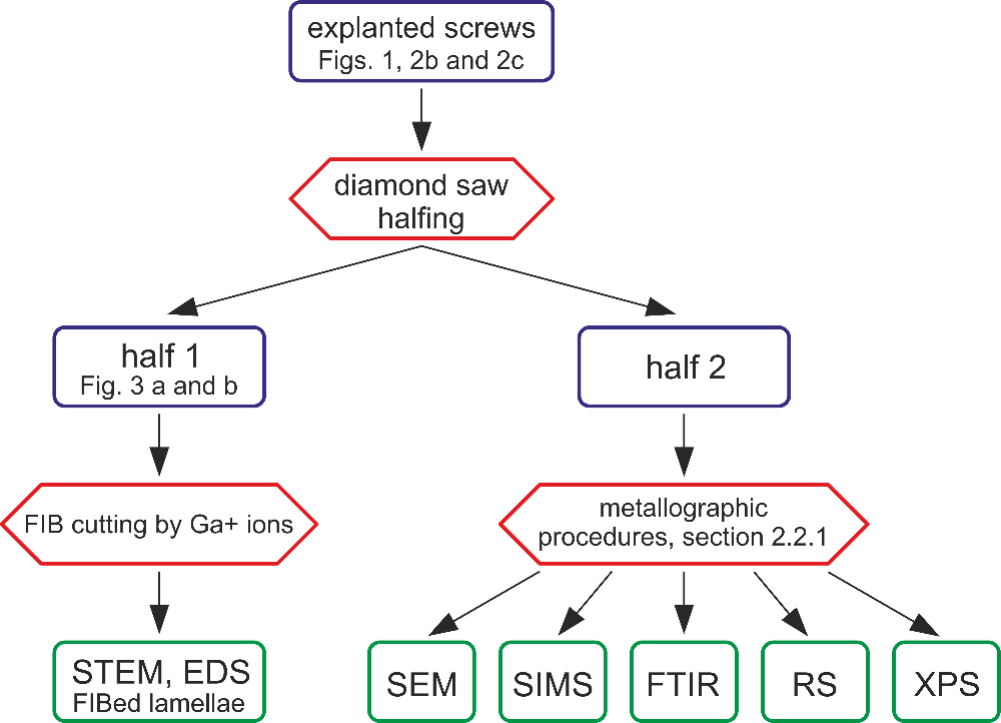
Schematic diagram summarizing the experimental procedure adopted in this study.

The overall experimental procedure is schematically illustrated by a flowchart shown in Figure 4. The scheme explains how the explanted material and its parts (blue delimited fields) were processed by cutting and metallography methods (red delimited fields) and the resulting samples were investigated by analytical techniques listed in the green fields.

## 3 Results

### 3.1 SEM Observations

As is demonstrated in the SEM images shown in Figure 2b,c, the cranial bone tightly fills spaces in the threaded structure. While in some locations the bone fully adheres to the steel and two such locations are marked by an arrow, gaps separating bone from the screw are also observed. The gaps result, most likely, from a shrinkage associated with the humidity loss which the bone tissue suffers during the sample preparation and during the exposure in the ultrahigh vacuum inside the SEM column.^30^

**Figure 5:**
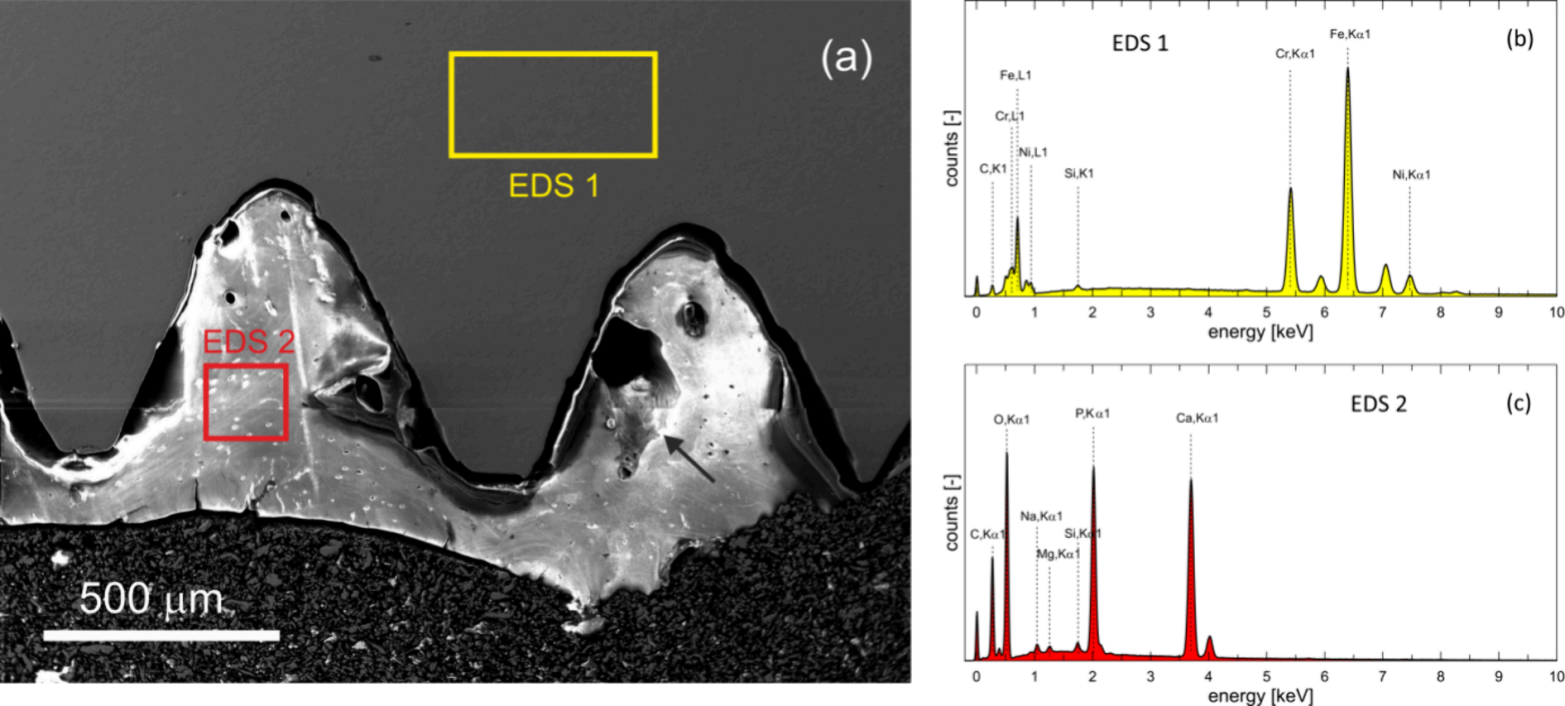
(a) Parts of the cortical cranial bone threaded in the steel screw, two rectangles (EDS 1 in the steel and EDS 2 in the bone) delimit areas from which the EDS signal was collected. The arrow indicates a resorption cavity. (b) EDS signal from the area EDS 1. (c) EDS signal from the area EDS 2.

The SEM image in Figure 5a characterizes the structure of the bone ingrown into two turnings of the screw. The fine topographic contrast in the secondary electron image reveals a lamellar arrangement of the bone forming regular osteons which are generally oriented parallel to the steel surface. A resorption cavity marked by an arrow results from osteoclast activity and suggests that standard bone remodelling takes place at the bone–steel interfaces throughout the period of the implantation. Numerous intersections between the canalicular system and the metallographic surface can be detected in the distant bone, while the canaliculi situated in close proximity to the steel–bone interface seem to be less frequent. Documentation related to the original surgery and thus information on the type of implanted material was not available anymore after 42 years. Therefore, global EDS data were collected from rectangular regions marked as EDS 1 in the implant (yellow) and EDS 2 in the bone (red). Corresponding EDS charts are shown in Figure 5b,c, and the resulting chemical compositions are listed in Table 1. The EDS results show that the screw was fabricated from the stainless steel AISI 304 while the analyzed region of the bone is well ossified containing an appropriate amount of calcium and phosphorus.

**Table 1:** Local Chemical Compositions (atom %) of the Steel Screw and Bone Acquired by EDS during SEM and STEM Observations

method	part	label/figure	C	Na	Mg	O	Si	P	Ca	Cr	Ni	Mn	Fe	total
SEM/area	steel	EDS 1/Figure 4	b	a	a	2.6	1.0	a	a	20.2	8.5	1.2	66.6	100.0
STEM/point	steel	EDS 3/c	b	a	a	1.6	1.0	0.1	0.0	19.8	8.5	0.9	68.1	100.0
STEM/point	steel	EDS 4/c	b	a	a	1.6	1.3	0.0	0.1	18.5	9.2	1.5	67.8	100.0
SEM/area	bone	EDS 2/Figure 4	b	1.0	0.5	79.1	0.4	8.8	10.2	a	a	a	a	100.0
STEM/point	bone	EDS 5/c	b	0.0	0.6	83.2	1.4	9.4	4.4	0.2	0.0	0.0	0.8	100.0
STEM/point	bone	EDS 6/c	b	0.0	0.0	71.7	4.8	11.4	9.2	0.5	0.0	0.1	2.3	100.0

a Not detected.

b Excluded due to hydrocarbon contamination.

c Figure not presented in this study.

### 3.2 Phases and Chemical Composition in Micro- and Nanoscale

#### 3.2.1 SIMS

**Figure 6:**
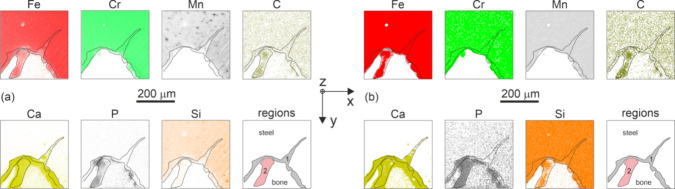
ToF-SIMS maps showing distribution of elements in the transition region between the AISI 304 screw and the cranial bone. Regions 1 and 2 represent, respectively, a steel–bone overlap over the 750 nm thick slice and a bone volume with an increased concentration of iron and silicon. (a) True integrated signal. (b) Posterized signal, see text for further details.

Figure 6 shows SIMS elemental maps collected in a region which covers one screw thread and the ingrown bone. As is commonly observed, the sputtering efficiency and related yield of single ions is lower in the bone part as compared to the metallic part.^31^ In Figure 6a, a true chemical signal is presented in the form of data which were integrated over an etching period between 5400 and 6000 s. Therefore, the integrated values represent an average chemical composition in a slice of material with an approximate thickness 750 nm situated in about 7.1 μm below the original metallographic surface. Apparently, this representation minimizes artifacts associated with potential near-surface contamination and with decay of the signal due to the increasing depth of the crater. We note that full depth profiles of Si^+^, Fe^+^, Cr^+^, and Mn^+^ cation signals are presented in the Supporting Information. In Figure 6b, the same SIMS signal was posterized in order to highlight locations with a prevalence of the individual elements. Due to irregularities of the interface and associated overlaps between the screw and the bone tissue, region 1 (gray) mixes the signal from both materials. Figure 6b clearly indicates that there is a region 2 (pink) inside the bone which, besides the common bone forming elements C, Ca, and P, also contains Fe and Si in relevant concentrations. These data suggest that the peri-implant bone may host complexes which mix iron and silicon together.

#### 3.2.2 Vibrational Spectroscopies

**Figure 7:**
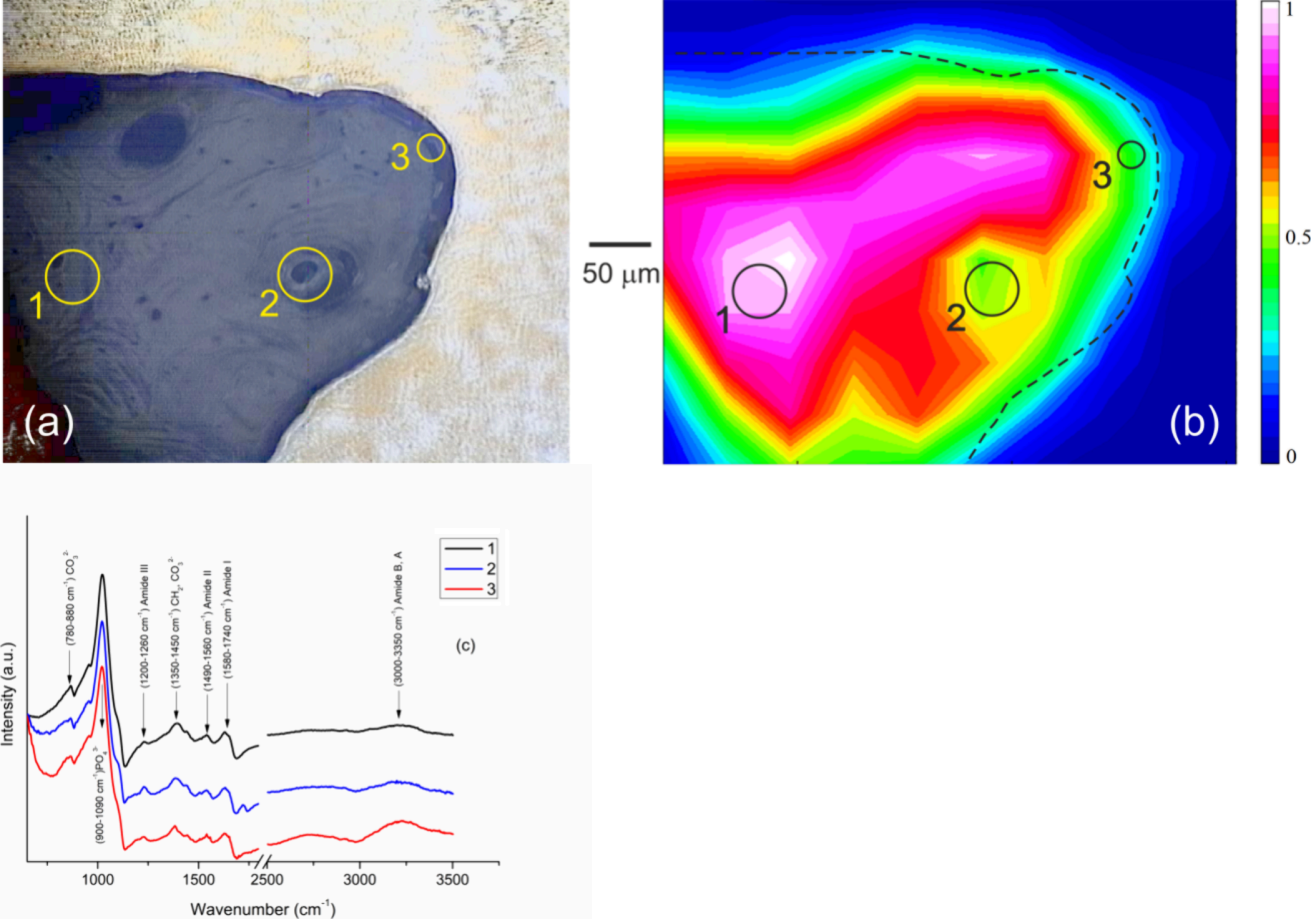
Results of FTIR analysis. (a) Light microscopy image shows the cortical bone (dark area) embedded in one thread of the AISI screw (bright area). Three regions are delimited by circles: 1 - distant bone, 2 - osteon, and 3 - peri-implant bone. (b) Intensity map of the integrated PO_4_^–3^ peak (960–1090 cm^–1^, see plot in panel c). (c) Spectral lines corresponding to positions 1, 2, and 3.

In order to assess the bone age in different positions relative to the bone–implant interface, FTIR experiments were performed with a particular focus on the PO_4_ phosphate group as a building block of hydroxyapatite. The bone mineralization through the hydroxyapatite content is known to increase with increasing age of the tissue.^32^ A dark area in the light microscopy image shown in Figure 7a represents the bone ingrown into one thread of the AISI screw (bright area). Three regions 1–3 delimited by circles represent, respectively, a distant bone (region 1), an osteon (region 2), and bone close to the interface (region 3). A map in Figure 7b covers the full area of Figure 7a and reveals excitations of phosphate ions ν_1_,ν_3_-PO_4_^3–^, see the corresponding peak situated between carbonate ν_2_-CO_3_^2–^ and Amide III peaks in the FTIR spectrum presented in Figure 7c. In the map, the integrated ν_1_,ν_3_-PO_4_^3–^ peak intensities were normalized by the overall FTIR signal in order to suppress changes in the absorbance due to local surface irregularities. As a result, the map in Figure 7b distinguishes mature bone regions, with relatively high PO_4_ signal and thus better mineralized collagen matrix, like a tissue in the position 1, from younger parts with lower PO_4_ content (the osteon in the position 2 and the bone region next to the interface). We note that the dashed line in Figure 7b, which represents the interface observed in the microscopy image shown in Figure 7a, is not fully consistent with the boundary between the bone and implant suggested by colors of the map. This is mainly due to a linear interpolation of the FTIR signal between individual measurement points which were grit-distributed over the bone part with a grit spacing of 30 μm.

Another measure which indicates the bone maturation state is given by the carbonate/phosphate ratio (C/P). In Figure 7c, both waggling CH_2_ and vibrational ν_2_-CO_3_^2–^ modes are present in the spectra between peaks of amides. With the aging of the mineral phase, the phosphate in hydroxyapatite crystals is substituted by carbonate and the C/P ratio grows.^33,34^ The C/P ratio was calculated from peaks 1020 cm^–1^ (phosphate) and 1410 cm^–1^ (carbonate) after subtracting the background which yielded values of 0.111, 0.106, and 0.099 for the distant bone (position 1), osteon (position 2), and peri-implant bone (position 3), respectively. With respect to the local age of the bone tissue, this result is consistent with the PO_4_ data presented in the map of Figure 7b. Moreover, organic/inorganic ratio was evaluated from peaks 1640 cm^–1^ (collagen, Amide I) and 1020 cm^–1^ (mineral, ν_3_-PO_4_^3–^). A high share of collagen fibers in the fresh osteon in the position 2 yields the highest organic/inorganic ratio of 0.126. This ratio decreases to 0.117 in the interface area and is the lowest in the distant bone (0.113). Consistent with the foregoing results, the bone tissue next to the interface exhibits the lowest mineralization. Finally, a detailed inspection of the FTIR spectra in Figure 7c shows that all the three investigated regions yield peaks from organic components Amide I (C=O and C–N stretch), Amide II, Amide III (both from N–H bending and C–N stretch), and the overtones of Amide II (Amide A and Amide B) coming from structure of type I collagen.^35,36^

**Figure 8:**
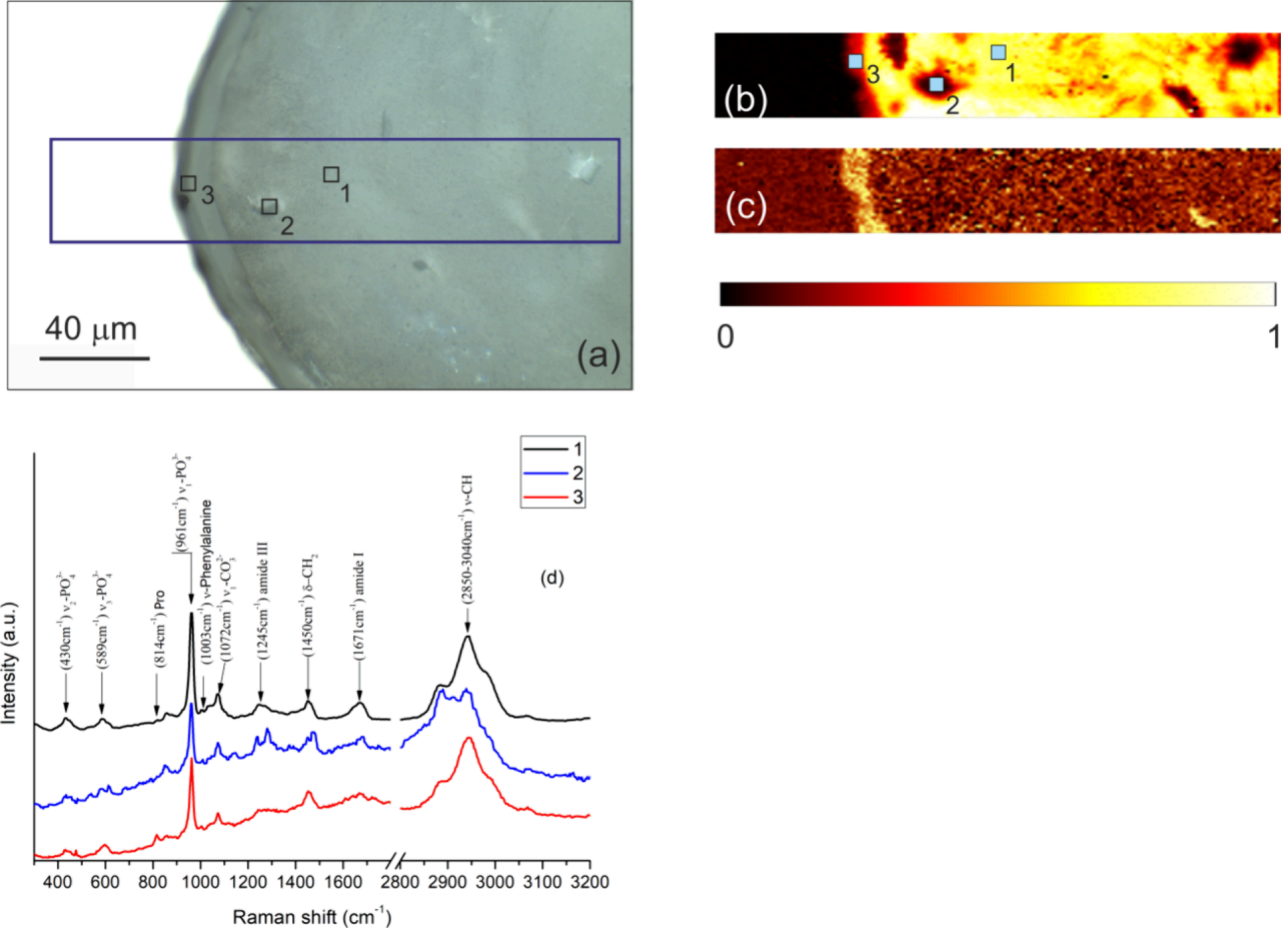
Results of Raman spectroscopy. (a) Blue rectangle in the light microscopy image marks the analyzed area in the bone (dark contrast) and in one thread of the AISI screw (bright contrast). Three regions are delimited by small squares: region 1 - distant bone, region 2 - lacuna, and region 3 - peri-implant bone. (b) An intensity map of integrated ν_1_-PO_4_^–3^ peak (961 cm^–1^, see plot in panel d). (c) An intensity map of the integrated Pro peak (814 cm^–1^, see plot in panel d). (d) Spectral lines corresponding to positions 1, 2 and 3.

An area investigated by Raman spectroscopy is delimited by a blue rectangle in the light microscopy image presented in Figure 8a. The area covers both the bone (dark) and screw (bright) parts with a particular focus on regions 1–3 marked by small squares. The normalized intensity of the phosphate peak at 961 cm^–1^ is mapped in Figure 8b; for the peak position see the spectra presented in Figure 8d. Similarly, the amino acid proline peak (Pro at 814 cm^–1^) gave rise to the map shown in Figure 8c. Consistent with the results of the FTIR spectroscopy, the distant bone in the position 1, with relatively high PO_4_ level in the map of Figure 8b, exhibits higher collagen mineralization as compared to other parts with lower PO_4_ content (like the lacuna in position 2 and the peri-implant bone in the position 3). This can be further documented by the local intensities in the indicated positions where the background-corrected heights of the ν_1_-PO_4_^–3^ peak are 0.84, 0.52, and 0.61 for positions 1, 2, and 3, respectively. Interestingly, the high intensity of the Pro peak in the interface region, see the map in Figure 8c, may indicate higher osteoblast activity since this amino acid is expected to promote osteoblast differentiation.^37^ Besides phosphate and Pro peaks, the three spectra in Figure 8d share many common peaks of inorganic and organic components, e.g., the ν_1_-CO_3_^–2^, δ-CH_2_, and amide I and III. Finally, an increase of residual fluorescence background observed for the lacunae in position 2 can be attributed to higher organic content, while the presence of iron ions may account for the same feature in the case of the interface spectrum in position 3.^38^

### 3.3 STEM

#### 3.3.1 SAD

**Figure 9:**
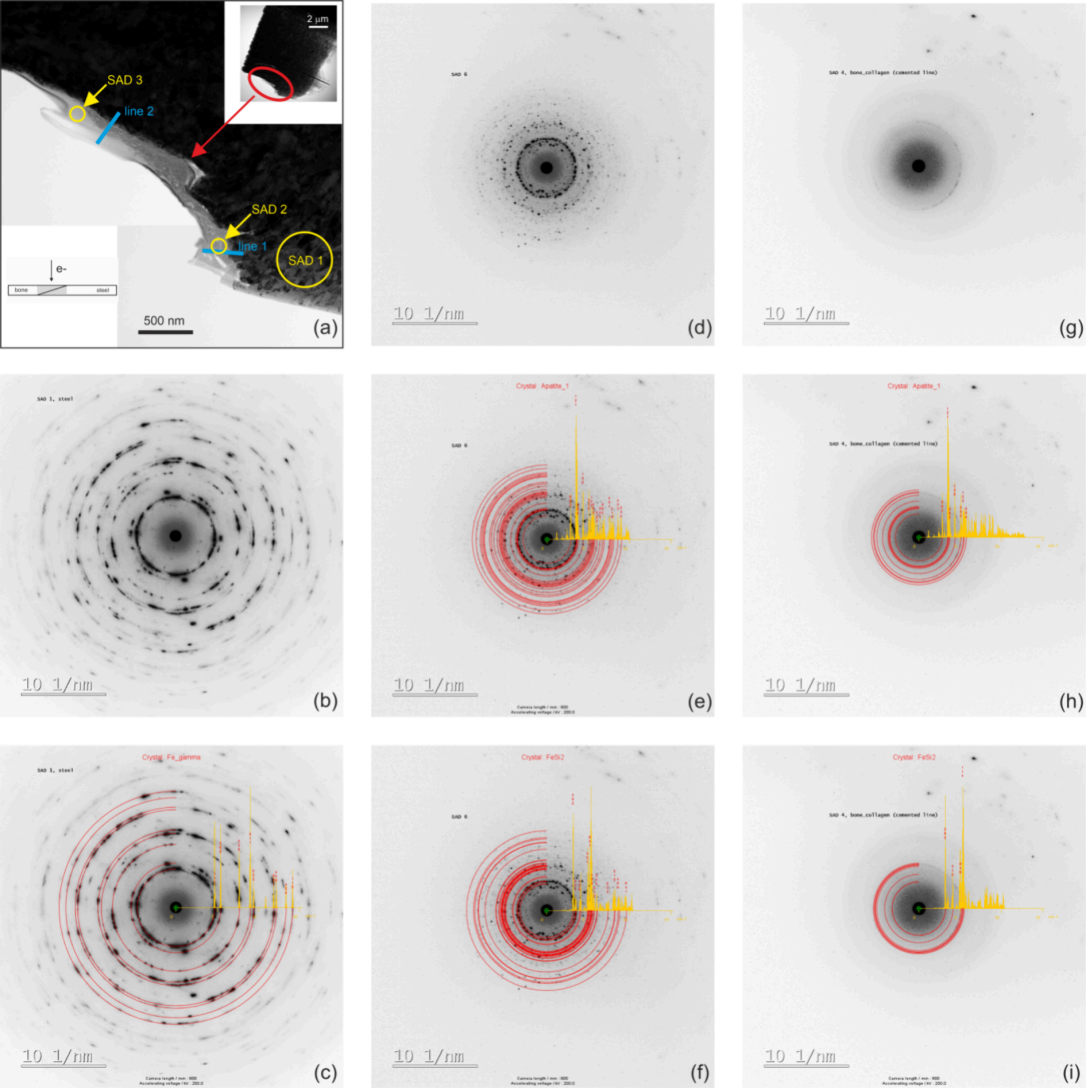
(a) STEM image of the FIBed lamella which covers the interface region between cranial bone and the AISI 304 steel screw. Positions of the SAD and EDS line experiments are indicted. (b) Experimental SAD1 from position 1 in the steel screw. (c) SAD1 complemented with JEMS-simulated diffraction rings (red). (d) Experimental SAD2 from position 2 in the calcified bone. (e, f) SAD2 complemented with JEMS-simulated diffraction rings (red). (g) Experimental SAD3 from position 3 in the uncalcified tissue. (h, i) SAD3 complemented with JEMS-simulated diffraction rings (red). See text for further details.

A thin STEM lamella shown in the upper right inset of Figure 9a was prepared by the FIB technique, see also Figure 3. The magnified view in Figure 9a zooms-in to the area delimited by the red ellipse where the bone tissue directly adheres to the AISI 304 steel implant. Yellow circles indicate positions of SAD experiments. We note that, as a result of processing conditions, the AISI 304 screws exhibit an ultrafine grain microstructure with a typical crystallite size on the order of 100 nm. Consequently, setting the aperture of 500 nm at the position SAD 1, the diffracted intensity forms the powder-like pattern presented in Figure 9b. JEMS simulations were performed based on the austenite structure file (ICSD no. 53449) in order to fit the experimental SAD 1 pattern with a set of calculated powder diffraction rings. Figure 9c documents an excellent match between the experimental intensities and the fitted rings (red). This procedure thus enabled an exact calibration of the TEM camera length, a step important for subsequent analyses of diffraction intensities collected from regions SAD 2 and 3. These experimental patterns are presented in Figure 9d,g, respectively. As can be seen in Figure 9a, SAD 2 was acquired from the calcified bone whereas SAD 3 originated from the yet collagenous region not enriched by the mineral components. The aperture size used for the diffractions SAD 2 and 3 was reduced to 150 nm in order to target exclusively these specific bone locations. Several inorganic phases were considered in order to compare experimental SAD 2 and 3 data to the corresponding JEMS simulations. Out of the testing set, only the two best fitting calculation outputs are shown in Figure 9e,f (SAD 2) and Figure 9h,i (SAD 3). These best fitting models were obtained for the hexagonal hydroxyapatite Ca_10_(PO_4_)_6_(OH)_2_ (Figure 9e,h, ICSD structure file no. 151939) and the orthorhombic FeSi_2_ phase (Figure 9f,i, ICSD structure file no. 9119). We can conclude that SAD 2 from the calcified bone can be satisfactorily accounted for by the hydroxyapatite model. Nevertheless, the orthorhombic FeSi_2_ model can also rationalize a considerable part of the SAD 2 diffracted intensity. Conversely, the situation is less clear for SAD 3 which exhibits features typical for an electron scattering from amorphous materials. Only one faint ring can be observed in Figure 9g which can be accounted for by (123) and (004) reflections from the hexagonal phase or, equivalently, by (040) and (114) reflections from the orthorhombic phase. This suggests that already the collagenous part of the bone may contain either some precursors of the hydroxyapatite or Fe–Si residues formed by ions resolved from the implant material during the remodelling cycles.

#### 3.3.2 EDS

**Figure 10:**
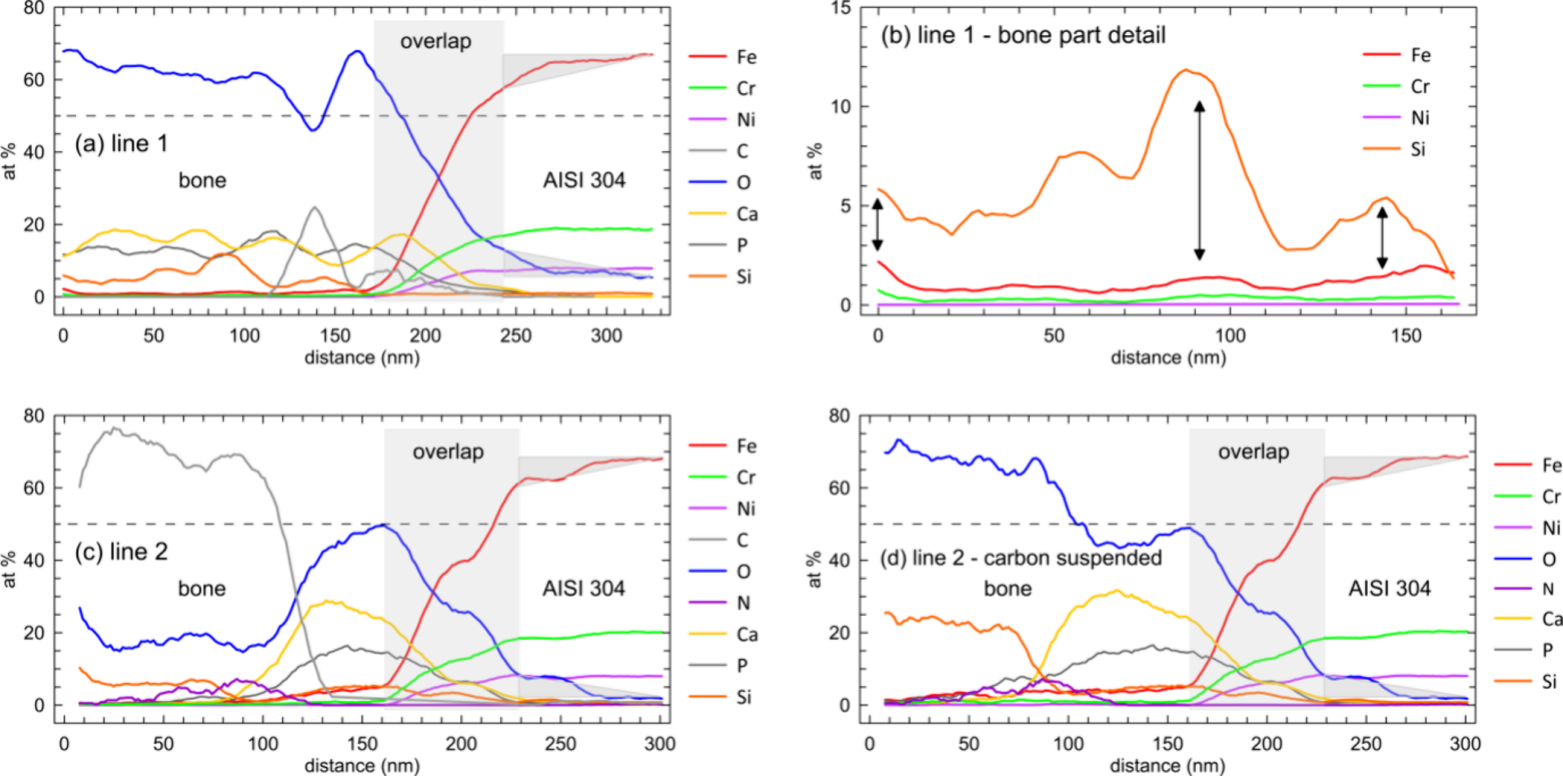
EDS line analyses taken across the bone–AISI implant interface. Positions of the individual lines are marked in Figure 9a. (a) Line 1 samples chemical composition in a transition between the implant and the calcified bone. (b) Magnified part of the EDS chart presented in panel a which focuses only on the bone region. Correlations between Si and Fe signals are highlighted by double-headed arrows. (c) Chemical composition recorded along line 2 taken through implant, calcified, and uncalcified segments of the bone. (d) Same chart as in panel c which better reveals the Si/O ratio under the condition of the missing carbon signal.

The SAD results presented in Figure 9 are consistent with local chemical compositions sampled by the EDS scans along lines 1 and 2 in Figure 9a, see the corresponding EDS charts in Figure 10. The chemical composition (atom %) recorded along line 1 is shown in Figure 10a. Here, oxygen is a dominant element in the bone part where also other elements typical for mineralized bone, namely calcium, phosphorus, and locally also carbon, are clearly detected. Slightly surprising is a rather high silicon content, locally exceeding 10 atom % and also a concentration of iron which oscillates in a range of 1–3 atom %. A region which mixes bone and steel compositions spans over about 70 nm and results from a bone–implant overlap schematically illustrated in the lower-left inset of Figure 9a. Finally, clear gradients of Fe and O in the steel part (highlighted by light-gray triangles) suggest that an approximately 80 nm thick steel layer has been modified by the bioenvironment attack and, at the same time, released some amount of iron ions in either Fe^2+^ (ferrous) or Fe^3+^ (ferric) form into the bone tissue.^24,25,39−47^Figure 10b shows a magnified part of the EDS chart acquired along line 1 in the bone only. Corresponding elemental distributions suggest that there is a correlation between silicon and iron signals, both elements accumulating in similar locations along the testing line; see the double-headed arrows in Figure 10b. Compositional differences between the implant, mineralized, and as yet unmineralized parts of the bone were sampled by line 2. High carbon content combined with oxygen and nitrogen in the unmineralized part of the bone points to the presence of collagen; see Figure 10c. However, in this collagenous tissue we have detected again a rather high concentration of silicon which is still present in the calcified part of the bone right at the interface and, similar to the line 1 analysis, correlates with the elevated signal of iron. The remaining features of the line 2 analysis, namely, the mixed signals over the overlap region and the Fe and O gradients in the steel implant, are fully comparable to the analysis along line 1. Finally, in order to assess a ratio between silicon and oxygen in the unmineralized part of the bone (line 2 analysis), we have suspended the carbon signal and plotted the result in Figure 10d. We note that the Si/O ratio is not far from 2 which may indicate presence of silica in the uncalcified bone matrix close to the interface.

### 3.4 XPS

**Figure 11:**
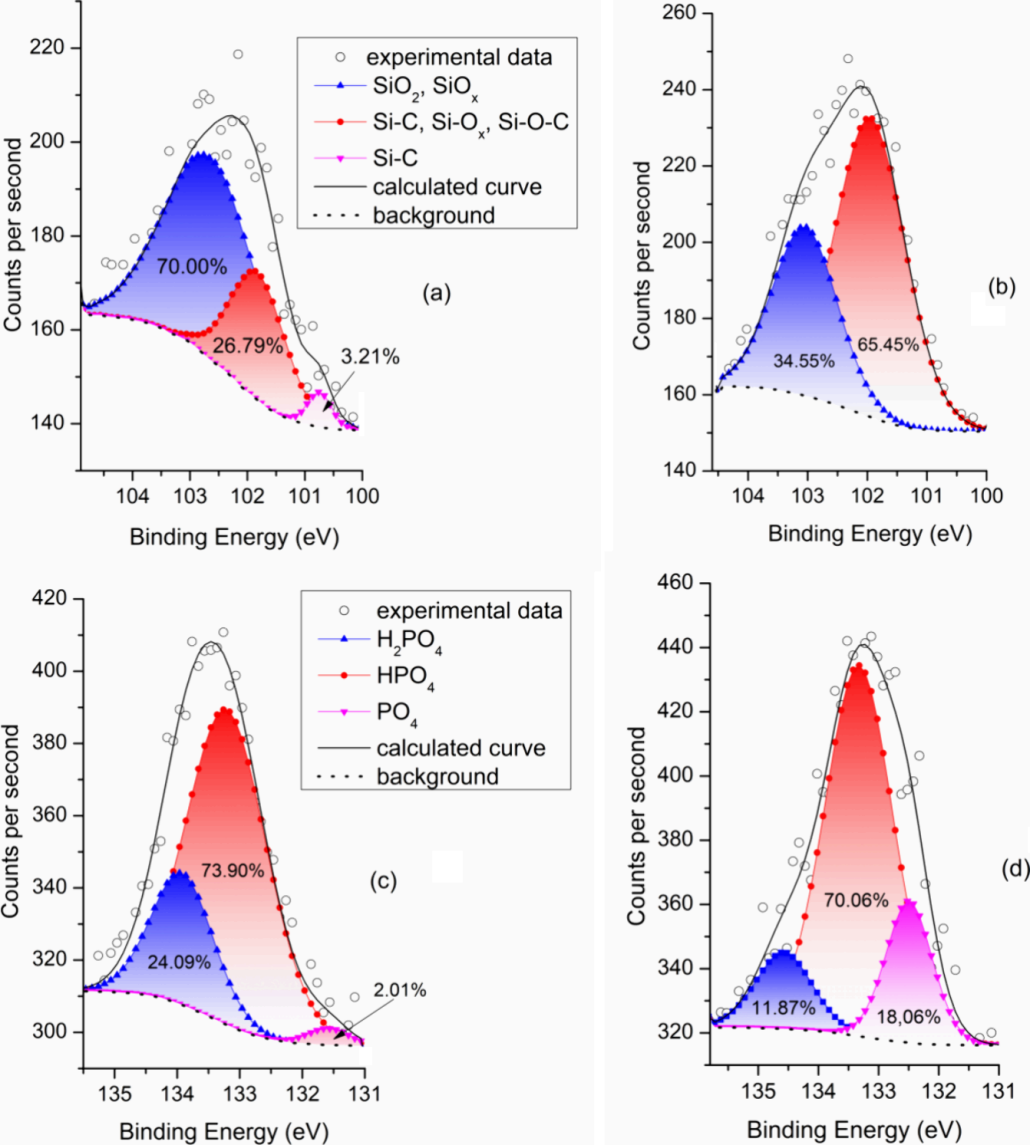
Deconvolutions of the XPS peaks based on characteristic binding energies and corresponding chemical environments. (a, b) Si 2p XPS signal from (a) interface and (b) distant bone. (c, d) P 2p XPS signal from (c) interface and (d) distant bone.

Since binding energies of electrons sensitively reflect chemical environments of atoms, XPS proved to be efficient at identifying chemical bonds in the bone tissue.^48^ Similar to the FTIR and Raman spectroscopies, the nature of silicon and phosphorus bonds was investigated by XPS in two locations, the distant and peri-implant bone. Consistent with the EDS data shown in Figure 10d, a deconvolution of the Si 2p peak presented in Figure 11a,b indicates that the peri-implant bone near the interface (Figure 11a) contains a considerably higher percentage of silica as compared to the bone area far from the interface (Figure 11b). This is in line with the reported effect of silica-substituted hydroxyapatite on the remodelling processes at the bone–implant interface.^49^ A deconvolution of the P 2p XPS peak shown in Figure 11c,d characterizes the bond state of phosphorus atoms in the implant–bone interface region (Figure 11c) and in the distant bone (Figure 11d).

**Figure 12:**
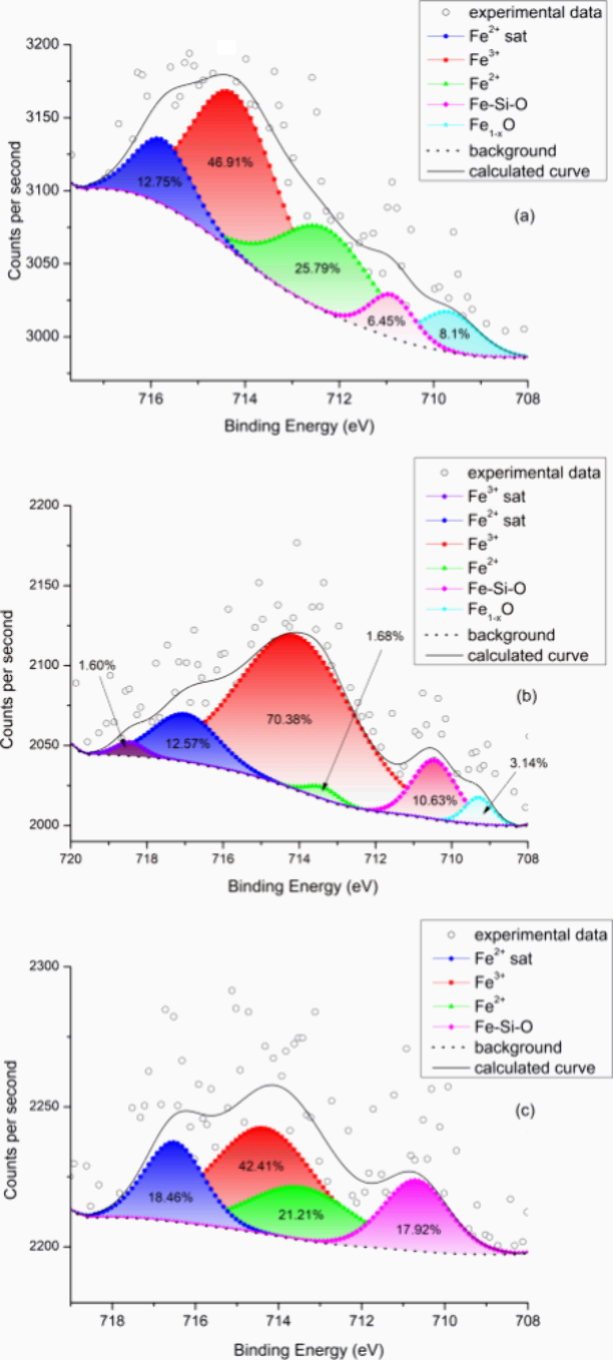
Ferrous and ferric iron ions were detected in all three locations investigated by the XPS technique: (a) explanted screw, (b) interface region, and (c) peri-implant bone. After deconvolution, the Fe 2p_3/2_ spectra show relative contributions of the individual ions and Fe–Si–O and Fe–O clusters.

The analysis of peaks reveals the presence and relative contributions of monohydrogen phosphate (HPO_4_^2–^), dihydrogen phosphate (H_2_PO_4_^–^), and phosphate (PO_4_^3–^) ions. As compared to the peri-implant bone, a higher content of PO_4_^3–^ ions in the distant bone indicates higher share of crystalline hydroxyapatite. In line with previous studies,^50^ a high signal due to the HPO_4_^2–^ ions in both regions suggests that the hydroxyapatite internal crystalline core is covered by an amorphous layer in which the HPO_4_^2–^ ions are concentrated. At the same time, the HPO_4_^2–^ ions promote buffering of blood together with H_2_PO^4–^ ions, which are all adsorbed by the implant surfaces during the contact with body fluids.^51^ Furthermore, XPS spectra shown in Figure 12 suggest that iron ions are present not only in the explanted screw (Figure 12a) but also in the bone section close to the implant interface (Figure 12b) and in the distant bone (Figure 12c). The iron atoms are involved in electron transport processes; they may outbalance the bone remodelling process in favor of the osteoclast mediated resorption which, under conditions of a strong iron overload, may result in bone loss and fractures.^41^ The Fe 2p_3/2_ spectra show both the presence of Fe^3+^ by the strong oxide peak at 714 eV at and evidence of Fe^2+^ ions by the peak at 712 eV and the satellite peak at 716 eV.^52,53^ A slight shift of peaks 710–711 eV to smaller binding energy at the interface area could belong to Fe–Si binary oxides.^54,55^ The peak at 709 eV corresponds to nonstoichiometric iron oxides.^56^

## 4 Discussion

The combination of FTIR, RS, and XPS analytical techniques used in the present study revealed systematically different bone chemical compositions close to and far from the implanted AISI 304 screws. All three spectroscopy methods independently, but consistently, detected a lower signal generated by the PO_4_ groups, and consequently lower bone mineralization,^32,34^ in a layer extending up to 50 μm from the implant surface. In what follows, we refer this bone portion as the interface bone layer (IBL). Incomplete mineralization was also detected in the osteon situated next to the IBL; see, e.g., Figure 7b. As is commonly acknowledged, increasing bone age scales positively with the PO_4_ level.^32,57,58^ Therefore, the systematically lower PO_4_ signal in the IBL indicates that these regions host, on average, younger bone tissue as compared to the bone parts situated distantly from the implant. This conclusion receives further experimental support both collected in the present study and presented so far in the literature. The data summarized in the section 3.2.2 document increasing carbonate-to-phosphate ratio with increasing distance from the interface. In line with the evidence presented in the literature,^33,34^ this confirms the local bone age distribution inferred from the intensity of the PO_4_ signal. Interestingly, similar variation in the local tissue age was reported by Shah and coauthors^59^ who investigated differences in a number of canaliculi per one osteocyte lacuna and found “less aged” tissue adjacent to the implant surface. Another signature indicating the lower bone age in the IBL was presented by the STEM image in Figure 10 showing as yet uncalcified tissue in direct contact with the implant. Finally, the higher intensity of the proline peak in the Raman spectra obtained from the IBL, see Figure 8, suggests higher activity of osteoblast cells and higher content of organic matrix.^19,37^

The bone segment age is critically dependent on the frequency of remodelling events in a particular bone location. A higher frequency of remodelling events is expected when either the bone tissue suffers from excessively high mechanical loading, generating damage in a form of microcracks, or contrarily, when it is in a “disuse state”.^60^ Apparently, neither of the two cases applies in the present study. First, the IBL tissue was hardly subjected to excessive mechanical loadings since it surrounded implants which rested quietly in the cranial bone for more than 42 years. Like the distant bone, the IBL does not exhibit any relevant signs of metallosis^61^ or an increased level of microdamage.^62^ Second, in a situation of excessively low mechanical loading (or the “disuse state”), the tissue in the IBL should be fully equivalent to the bone far from the interface. In such a situation, the higher frequency of remodelling events, and thus younger bone, would be observed not only in the IBL but also farther from the interface.

The resorption part of the remodelling cycle mediated by osteoclast cells is associated with an acidic attack which accelerates the transition of the metallic ions from the steel screw into the bone tissue.^24^ During long-term implantation, individual surface locations of the screws experience several local remodelling events and thus are exposed to several acidic attacks. Their average number and time *T*_c_ between two bone remodelling cycles at the same interface position can be estimated based on the thickness of the implant layer, *h*_exp_ = 8 × 10^–8^ m, affected by corrosion, see Figure 10. Pardo et al. investigated AISI 304 steel corrosion in 3.5 M H_2_SO_4_ (pH of −0.85) at 25 and 50 °C.^63^ Their data yield a rate of 3.18 × 10^–4^ m day^–1^ by means of which the affected surface layer grows at 37 °C. We assume that the weak biocorrosion of the AISI 304 implant is mainly due to direct contact established between osteoclast annular sealing zones and the implant surfaces during the bone resorption periods, see Figure 5a. With respect to the moderate acidity inside the sealing zones (pH about 5, see refs (64 and 65)), the corrosion rates reported by Pardo et al. must be modified using a ratio 1.43 × 10^–6^ between molarities of H^+^ ions in the osteoclast compartments and in Pardo’s solution.^63^ As a result, the corrosion rate caused by direct contact between multinucleated osteoclast cells and the implant surface is expected to decrease to *ḣ*_corr_ = 4.55 × 10^–10^ m day^–1^. In their review, Kenkre and Bassett^66^ summarized data on time intervals which characterize individual phases of the bone remodelling cycle. In particular, they indicated that the bone resorption phase typically requires a time period of *h*_res_ = 14 days. Knowing these parameters, an average number *N*_rem_ of remodelling events occurring at a particular interface location over the total time of *N*_tot_ = 42 years during which the implant rested in the cranial bone can be estimated from the equation
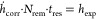
1which yields *N*_rem_ = 12.6. Finally, the average time period *T*_c_ can be calculated as *T*_c_ = *T*_tot_/*N*_rem_ = 3.3 years. In passing, we note that these estimates are in very good agreement with the data yielded by the statistical analysis, see section 3 of the Supporting Information. Therefore, we anticipate that the higher remodelling frequency is associated with mild corrosion of the AISI 304 steel implants and related influx of metallic ions into the IBL.

Experiments with iron overloaded mice indicated the high bone turnover, which was present in both males and females in a range of animal ages.^67^ Our SIMS and EDS analytical techniques clearly documented an accumulation of metallic elements, particularly iron, in the peri-implant bone. In the same time, the high-resolution EDS revealed the increased oxygen level in an approximately 80 nm thick interface layer of the implant, see Figures 6 and 10. There is ample evidence in the literature showing that an excess of iron ions stimulates osteoclast precursor proliferation and differentiation and the activity of mature osteoclasts leading to accelerated bone resorption.^42,68−70^ In this respect, in vitro studies have shown that human osteoclasts can corrode stainless steel leading to the production of metal ions^24^ while traces of cellular activities are left behind on surfaces of metal orthopedic explants.^25^ The osteoclast cells are known to increase the acidity within their extracellular compartments to pH about 5, using proton pumps located in the basolateral membrane.^64,65^ Some of the extracellular compartments establish direct contact with the implant surface and thus accelerate considerably the transfer of Fe^*n*+^ ions into the bioenvironment. Under an assumption that concentration gradients drive migration of the metallic ions through the bone tissue,^71^ we have formulated a simple model which accounts for the accumulation of the ions in the IBL; see section 4 in the Supporting Information. As can be shown by the integration of concentration profiles plotted in Figure S5, nearly 60% of the metallic ions dissolved at the resorption site during one hour remain in the approximately 150 μm thick interface bone layer. A time domain of one hour seems to be sufficient for significant transport of iron ions into the interior of cells.^72^

Considering all the experimental data and the numerical results, we suggest that the higher turnover rate in the IBL is governed by an autocatalytic process in which the higher concentration of Fe^*n*+^ ions released from the implant stimulates the osteoclast activity while the associated higher number of fresh resorption sites, in turn, promote implant corrosion and enrich the IBL with a surplus of Fe^*n*+^ ions. A brief estimate suggests that the IBL underwent between 12 and 13 remodelling cycles during the 42 year implantation of the AISI 304 screws. Since this would correspond to about a 3 year turnover period, we may conclude that the turnover of the peri-implant bone was accelerated to about three times with respect to the cranial bone far from the implant. The calculation was based on known corrosion rates of AISI 304 steel^63^ and the thickness of the implant layer enriched in oxygen as detected by the EDS technique, see Figure 10. Concerning the iron transfer from the implant into the bone tissue, another interesting experimental result presented in Figure 10b indicates a correlation between chemical signals of iron and silicon. Since silicon is an essential mineral for bone formation^73,74^ and, at the same time, exhibits a high affinity to both iron^75^ and titanium,^76^ the formation of Fe–Si complexes in the bone tissue (Figure 6) can be expected. Nevertheless, a more general role of bone silicon in the corrosion of metallic implants would require further targeted investigation.

## 5 Summary and Conclusions

A selection of spectroscopy and electron microscopy techniques was employed in order to characterize interfaces that were formed during 42 years of interaction between cortical cranial bone and navigation screws made of AISI 304 stainless steel. Results of the experimental analyses and simple numerical models suggest that the peri-implant bone layer was subjected to accelerated turnover with a characteristic period of about 3 years. The drop in the period between consecutive remodelling events can be accounted for by an autocatalytic process in which the higher concentration of Fe^*n*+^ ions released from the implant stimulates the osteoclast activity while the associated higher number of fresh resorption sites, in turn, promote implant corrosion and enrich the IBL with a surplus of Fe^*n*+^ ions. A comparison of data acquired from the peri-implant bone (the interface bone layer IBL) and the cranial bone situated far from the implant (distant bone DB) supports the following main conclusions:

(1) The FTIR and Raman spectroscopy signals due to PO_4_^3–^ groups increase along the path from IBL toward DB.
(2) The carbonate to phosphate ratio is lower while the organic to inorganic ration is higher in the IBL. The Raman Pro peak was only detected in the IBL.
(3) The XPS signal from silica is high and, consistent with the FTIR and Raman results, the PO_4_^3–^ peak is suppressed in the IBL.
(4) SIMS measurements, TEM diffraction, and high-resolution EDS independently detected Fe–Si complexes in the IBL.
(5) The high-resolution EDS analyses confirmed penetration of oxygen into the implant surface where it forms up to 80 nm thick oxygen-enriched interface layers. This can be primarily accounted for by biocorrosion associated with an acidic attack during remodelling cycles.
(6) The presence of generally less aged tissue in the IBL would consistently rationalize experimental data and numerical results reported in the present study.

While accelerated bone turnover has traditionally been attributed to either excessively high mechanical loadings and related damage in the form of microcracks or contrarily to a “disuse state”,^60^ results of the present study suggest that the acceleration can also be stimulated by chemical effects. Specifically, this study establishes a link between the accelerated turnover in the peri-implant bone area and the Fe^*n*+^ ions released from the AISI 304 cranial screws due to long-term mild corrosion.

## Supporting Information Available

(i) Additional details on the experimental methodologies, (ii) a complete SIMS chemical profile, (iii) probabilistic calculation of bone remodelling events, and (iv) 2D diffusion model addressing distribution of metallic impurities in the bone tissue (PDF)
