# Supplementary material for: Interfaces between Cranial Bone and AISI 304 Steel after Long-Term Implantation: A Case Study of Cranial Screws
Source: ACS Biomater Sci Eng. 2024 Jun 20;10(7):4297–310. doi: 10.1021/acsbiomaterials.4c00309 (PMC11234332; doi:10.1021/acsbiomaterials.4c00309)
Supplement: Supplementary file 1 — ab4c00309_si_001.pdf [file ab4c00309_si_001.pdf]

Manuscript title:

**Interfaces between Cranial Bone and AISI 304 Steel after Long-Term Implantation - A case study of cranial screws**

Authors:

Natália Luptáková[1], Václav Dlouhý[2], Dinara Sobola[1], Stanislava Fintová[1], Adam Weiser[1], Vladimír Beneš 3rd[2], Antonín Dlouhý[1*]

Affiliations:

[1] Institute of Physics of Materials, AS CR, v. v. i., Žižkova 513/22, Brno, 61662, Czech Republic

[2] Department of Neurosurgery, Second Faculty of Medicine, Charles University and University Hospital Motol, V Úvalu 84, Prague, 150 06, Czech Republic

*corresponding author: Antonín Dlouhý, Institute of Physics of Materials, AS CR, v. v. i., Žižkova 513/22, Brno, 61662, Czech Republic, dlouhy@ipm.cz

Number of pages: 8

Number of figures: 5

Number of tables: 1

Number of references: 7

## 1. Additional details on the experimental methodologies

For XPS studies of interfaces, it is essential to utilize an aperture (in our case, 110 μm in diameter) to improve spatial resolution. This is because the default spot size of XPS analysis (slot mode) is 200x700 μm. Increasing the accumulation numbers up to 15 sweeps, focusing on the most poorly detected element, helps mitigate the influence of noise.

SIMS was conducted as the final analysis step since we employed a dynamic (destructive) mode. To mitigate matrix effects and other artifacts, we adjusted the sputtering rate and introduced 1-second delays between sputtering and analysis to stabilize molecular dynamics on the surface of the interface. Additionally, we conducted long-term analysis of surface composition to ensure a reliable signal for the time-of-flight detector. During both SIMS and XPS analyses, the samples were securely fastened to the stage using conductive fixtures on all sides to prevent charging effects, with the exception of the area being analysed.

Prior to making measurements and drawing conclusions regarding phonon spectra for distinct chemical bonds, both Raman and FTIR instruments were meticulously calibrated to ensure precise peak positions. The Raman spectroscope was calibrated using a Si wafer with a native $SiO_2$ layer, while the FTIR was calibrated using thin gold films. Vibrational spectroscopies did not reveal peaks for the metallic material of the navigation rod.

The Raman mapping duration was 20 hours, 10 minutes, and 6 seconds to maintain an optimal signal-to-noise ratio. A long integration time (12 seconds) was utilized for obtaining Raman spectra to minimize spurious features such as cosmic rays. Additionally, special attention was given to accumulation numbers (256) to reduce the influence of random noise effects after a 30-minute temperature stabilization of the nitrogen-cooled MCT detector.

The measurements were conducted in an ISO Class 8 (US FS 100,000) cleanroom environment.

## 2. SIMS

SIMS data covering 12000 s, that is the total time and depth range of the SIMS experiment (see the corresponding x-y maps of a selected time slice shown in Fig. 6 of the main article), are presented in Fig. S1. For each investigated element, the signal intensities were depth-integrated and converted into gray level images. Subsequently, the values of individual pixels were divided by 256 and thus transformed into the (0,1) interval. This normalization facilitates a comparison of SIMS signals that may otherwise exhibit order of magnitude differences. The resulting normalized signals of individual elements are shown in Fig. S1a, which illustrates the full depth elemental profiles at y = 260 μm plotted along the x-direction. Figure S1b presents the depth vers. x-coordinate concentration maps of Si, Fe, Cr and Mn ions obtained at the same y-level of 260 μm. Both representations of the SIMS chemical data provide additional evidence for the formation of Fe-Si clusters in the bone tissue.

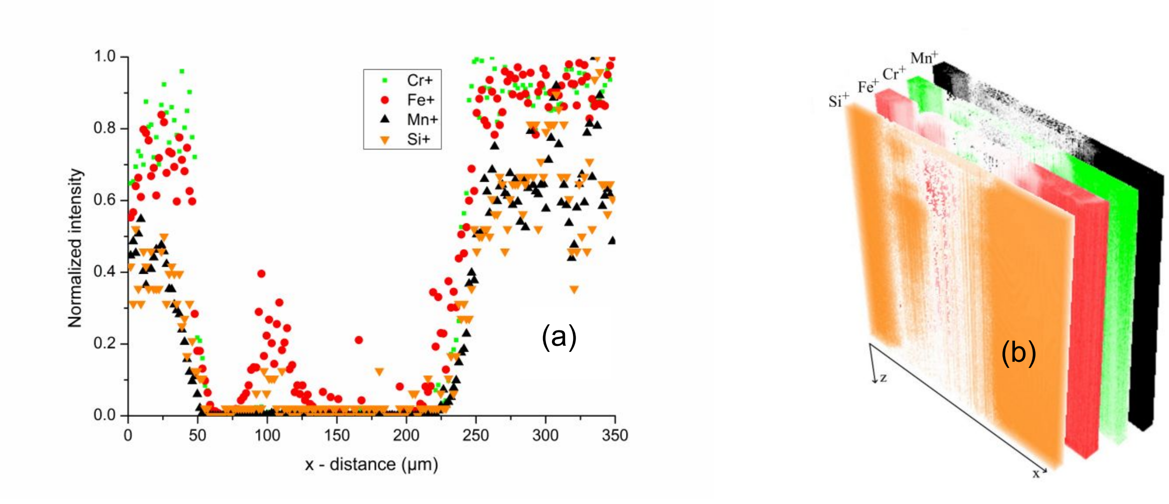


Fig S1. (a) A slice taken at y=260 μm represents full depth-integrated chemical profiles plotted along the x-direction. (b) Concentration maps of Si, Fe, Cr and Mn ions in the plane (y=260 μm) delimited by the depth and x-coordinate. See text for further details.

## 3. Remodelling events and their probabilities

The experimental evidence (Fig. 5 of the main article, where the resorption cavity was documented), shows that the bone remodelling processes take place at the AISI 304 - bone interface throughout the period during which the implant rests in the cranial bone. Similar remodelling processes at the bone-implant interfaces were reported in the literature [S1], but

their frequency at a particular interface location, which is of interest, remains essentially unknown. In order to provide some quantitative estimates, we have adopted a statistical approach based on normally distributed bone turnover periods $t_{C,i,j}$. Each period represents a time span between two consecutive bone remodelling events. Altogether, the statistics addresses a sample of $N$ bone locations (compartments or moieties [S2]) indexed by $i$. In each location $i$ we consider a series of $M$ consecutive remodelling cycles which distribute the compartments into $M$ age groups ("offsprings") indexed by $j$. We assume that the distribution of the turnover periods $t_{C,i,j}$ can be characterized by a mean $T_C$ and corresponding standard deviation $\delta_C$. Considering time point $t_R$ when the implant is removed from the cranial bone, the calculations focus on a number of accumulated remodelling events and a characteristic age of the bone tissue at a position $i$.

In a first step, the model starts off with the bone age $a_{ini,i}$ in each $i$-th compartment at the instant of the implant insertion $t_{ini} = 0$. The ages $a_{ini,i}$ are also normally distributed with a mean $T_{ini}$ and standard deviation $\delta_{ini}$. After the implantation, the original bone still remains in place for time $\Delta_i = T_B - a_{ini,i}$, where $T_B$ is a mean turnover period of the distant bone far from the implant. If the quantity $\Delta_i$ turns out negative, then $\Delta_i$ is taken as zero. After the time $\Delta_i$, the original bone in the $i$-th compartment is replaced by a new tissue as a result of the first remodelling event. This new tissue then represents the first "offspring" ($j = 1$) in the compartment $i$. The bone age evolution in each compartment thus can be characterized using consecutive accumulations of the turnover periods

$$t_{i,m} = \Delta_i + \sum_{j=1}^{m} t_{C,i,j} \ , \ m = 1,...,M \tag{SM1}$$

The implant is removed from the cranial bone at time $t_R$ and we seek for instances when the accumulated turnover periods in the compartment $i$ fulfil a condition

$$t_{i,m-1} < t_R < t_{i,m} \ , \ m = m_{R,i} \tag{SM2}$$

for a specific offspring $m = m_{R,i}$. For a sufficiently large number of the remodelling cycles M, this condition holds true in each compartment since the bone is still present in each compartment at the removal time. The model thus yields the required data in terms of the bone

age $a_{R,i} = t_R - t_{i,m_{R,i}-1}$ and the number of corresponding remodelling cycles $m_{R,i}$ which took place till the implant removal.

In order to estimate the key parameters, we have considered different mean turnover periods $T_C$ covering a range between 9 years, which is expected for the distant cortical bone [S3, S4], down to 3 years, which may reflect a possible acceleration of the remodelling cycles at the bone-implant interface. The model input parameters used for these calculations are listed in Table S1. Figure S2a shows how the bone age at the time of implantation $T_{ini}$ influences the number of offsprings $\bar{m}_R$ needed for the bone survival till the time of implant removal. The number of offsprings $\bar{m}_R$, which also represents an averaged number of remodelling cycles accumulated till the explantation, is calculated as a weighted average $\bar{m}_R = \sum_r m_r \cdot n_r / N$ of all specific $m_r$s that fulfil the condition Eq. 2 while the $n_r$s count compartments with the specific $m_r$. We note that $\sum_r n_r / N = 1$ and the error bars represent standard deviations $\delta_{\bar{m}_R} = \delta_C \cdot \sqrt{\sum_r n_r / N}$.

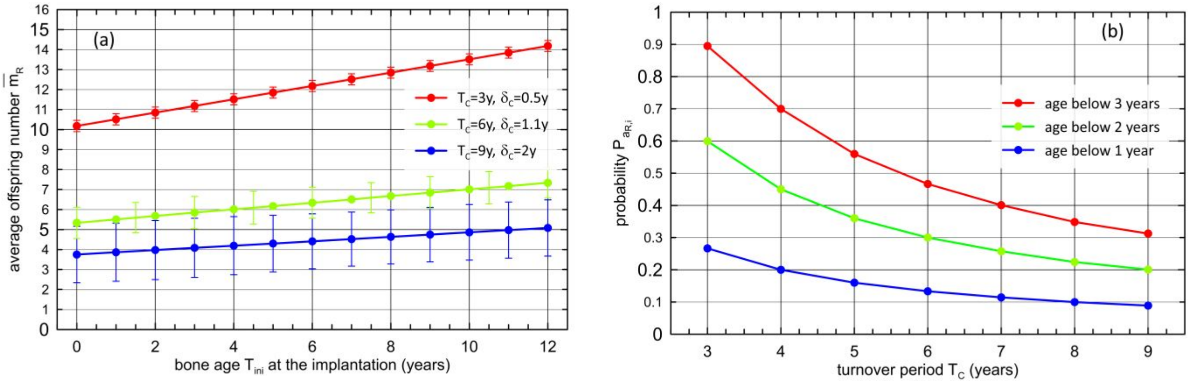


Figure S2: (a) A number of bone remodelling cycles accumulated till the explantation plotted against the age of the bone Tini at the instant of the implantation. The three lines correspond to three different frequencies of the remodelling cycle, TC = 9 years (blue), TC = 6 years (green) and TC = 3 years (red).

(b) Probability lines represent a likelihood that a bone in one particular compartment (out of 106 compartments) would be younger than 1 year (blue), 2 years (green) and 3 years (red). The probabilities are plotted as a function of the turnover periods which represent a characteristic time between two consecutive remodelling events.

As expected, the accelerated turnovers (lower $T_C$ values) result in a higher number of offsprings required to take the bone tissue through to the time point when the implant is removed, see the three dependences in Fig. S2a parametrized by the $T_C$ value. With respect to the accumulation of cations released from the implant into the neighbouring bone tissue, we are concerned about

an expected age of the bone which is in contact with the steel implant surface at the instant of implant removal. Figure S2b summarizes $P_{a_{R,i}}$ values which represent a probability that the bone tissue in a selected compartment would be younger than either 1 or 2 or 3 years. As it is evident from the plot, the probabilities clearly increase on the acceleration of the bone turnover. In contrast to the $\overline{m}_R$ numbers, the probabilities are almost independent of the bone age $T_{ini}$ at the time of implantation, see Fig. S3.

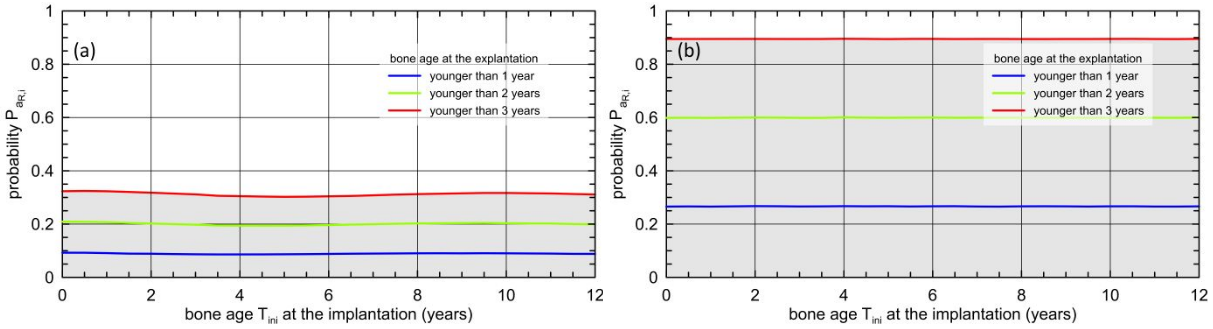


Figure S3: The probability lines represent a likelihood of finding a bone part (compartment) which is, at the instant of explantation, younger than 1 (blue), 2 (green) and 3 (red) years. Note that the probabilities change considerably with the frequency of remodelling cycles: (a) $T_C$ = 9 years, (b) $T_C$ = 3 years, but are almost independent of the bone age $T_{ini}$ at the instant of the implantation.

**Table S1** Parameters used as an input for statistical calculations.

| Calculation No. | $T_C$ (years) | $\delta_C$ (years) | $t_R$ (years) | $T_{ini}$ (years) | $\delta_{ini}$ (years) | $T_B$ (years) | $\delta_B$ (years) | N | M |
|---|---|---|---|---|---|---|---|---|---|
| 1 | 3 | 0.5 | 42 | 0-12 | 2 | 12 | 2 | $10^6$ | 20 |
| 2 | 4 | 0.5 | 42 | 0-12 | 2 | 12 | 2 | $10^6$ | 15 |
| 3 | 5 | 0.8 | 42 | 0-12 | 2 | 12 | 2 | $10^6$ | 15 |
| 4 | 6 | 1.1 | 42 | 0-12 | 2 | 12 | 2 | $10^6$ | 15 |
| 5 | 7 | 1.4 | 42 | 0-12 | 2 | 12 | 2 | $10^6$ | 10 |
| 6 | 8 | 1.7 | 42 | 0-12 | 2 | 12 | 2 | $10^6$ | 10 |
| 7 | 9 | 2 | 42 | 0-12 | 2 | 12 | 2 | $10^6$ | 10 |

## 4. Diffusion-driven distribution of metallic impurities in the bone tissue

Here we provide a semiquantitative insight into a spreading of metallic ions throughout the bone tissue. We assume that a source of the ions is a bone resorption site (RS) situated at the bone-metallic implant interface and that the ion propagation through the bio-environment is driven by their concentration gradients [S5]. Within this framework, we numerically solve the equation

$$\frac{\partial c}{\partial t} = D \cdot \left( \frac{\partial^2 c}{\partial x^2} + \frac{\partial^2 c}{\partial y^2} \right) \tag{SM3}$$

in an array of 40 x 30 square elements with dimensions of 25 x 25 $\mu m^2$ and sides parallel to $x$ and $y$ directions. The solution in terms of a normalized concentration $c(x, y, t)$ of the metallic ions acquires values from the interval <0,1>, fulfils periodic boundary conditions along the $x$ direction and assumes zero $y$-component of the ion flux $\frac{\partial c}{\partial y}(x, y_i, t) = 0$ at the interface ($y_i = 0$) and at the upper boundary of the region ($y_i = 750\,\mu m$). The initial condition sets $c(x, y, 0) = 1$ in the RS and $c(x, y, 0) = 0$ in the rest of the region. With respect to the objectives of these calculations, details of the ion migration mechanism are of lesser importance, therefore, we fix the diffusion coefficient at a value $D = 10\,\mu m^2\ s^{-1}$ [S6]. Calculation results are first presented in a form of concentration maps in Fig. S4. The maps characterize the evolving distributions of metallic ions at times t = 0, 600, 1800 and 3600 s. Similarly, plots in Fig. S5 show how the relative concentration profile along the x-direction (Fig. S5 a) and the y-direction (Fig. S5 b) evolves with increasing time. The profiles were obtained by cuts at $y_{cut} = 112.5\,\mu m$ (Fig. S5 a) and $x_{cut} = 62.5\,\mu m$ (Fig. S5 b) and clearly show that, within the investigated time domain, a majority of the metallic ions accumulates in about 150 μm thick layer in contact with the implant surface.

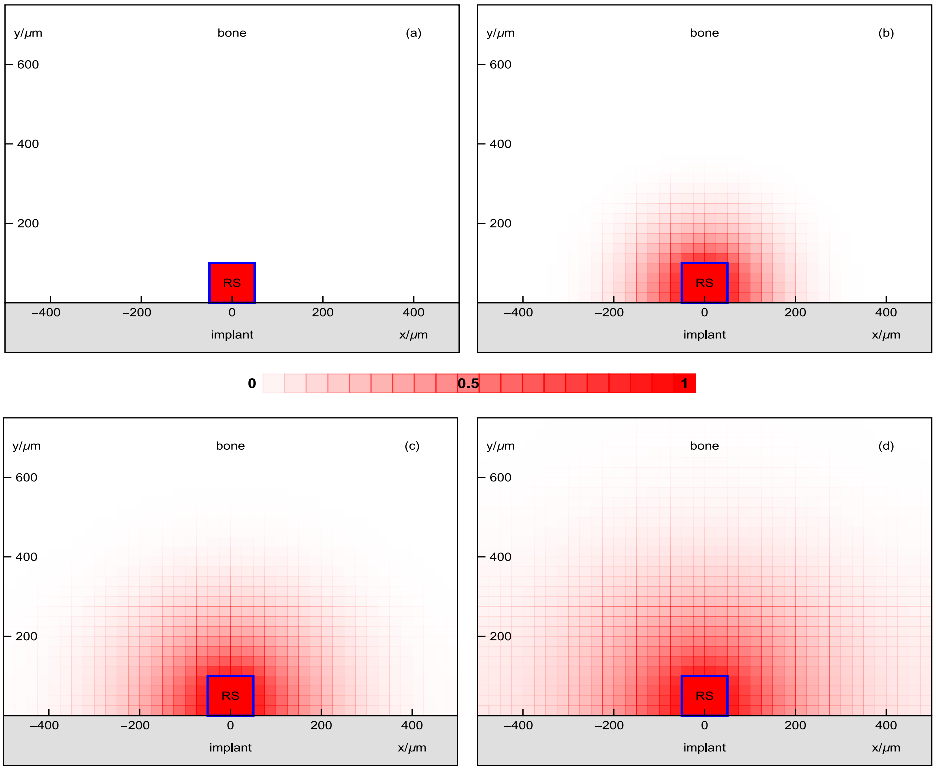


Figure S4: An intensity of red colour represents a relative concentration $c(x, y, t)$ of metallic ions in the bone tissue containing the resorption site RS (marked by a blue square, $c_{RS}(t) = 1$) at the bone-implant interface. Snapshots taken at (a) 0 s, (b) 600 s, (c) 1800 s and (d) 3600 s.

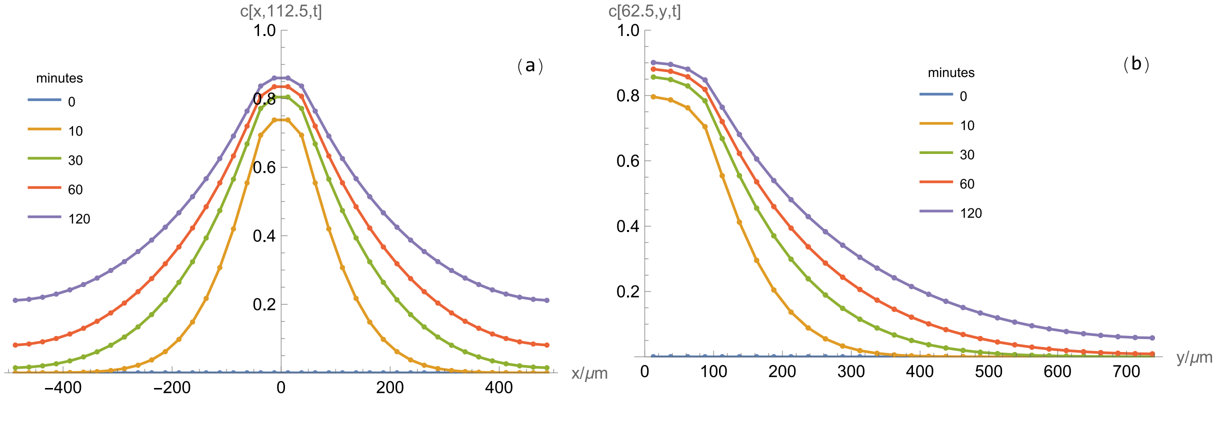


Figure S5: Profiles of the relative concentration $c(x, y, t)$ plotted at $t$ = 0, 600, 1800, 3600 and 7200 s. (a) *x*-profile obtained at $y_{cut} = 112.5\,\mu\text{m}$, (b) *y*-profile obtained at $x_{cut} = 62.5\,\mu\text{m}$.

**References**

[S1] Shah et al., High-resolution visualisation of the osteocyte lacuno-canalicular network juxtaposed to the surface of nanotextured titanium implants in human, ACS Biomater. Sci. Eng. 1 (2015) 305-313.

[S2] A.M. Parfitt, Misconceptions (2): Turnover Is Always Higher in Cancellous Than in Cortical Bone, Bone 30, (2002) 807-809.

[S3] S.C. Manolagas, Birth and Death of Bone Cells: Basic Regulatory Mechanisms and Implications for the Pathogenesis and Treatment of Osteoporosis, Endocrine Reviews 21 (2000) 115-137.

[S4] S. Qiu, D.S. Rao, S. Palnitkar, A.M. Parfitt, Age and Distance from the Surface but Not Menopause Reduce Osteocyte Density in Human Cancellous Bone, Bone 31 (2002) 313-318.

[S5] M.B. Schaffler, W.Y. Cheung, R. Majeska, O. Kennedy, Osteocytes: Master Orchestrators of Bone, Calcif Tissue Int 94 (2014) 5–24, doi: 10.1007/s00223-013-9790-y.

[S6] A. Bayani1, J.L. Dunster, J.J. Crofts, M. R. Nelson, Mechanisms and Points of Control in the Spread of Inflammation: A Mathematical Investigation, Bulletin of Mathematical Biology (2020) 82:45, https://doi.org/10.1007/s11538-020-00709-y.

[S7] J.S. Kenkre, J.H.D. Bassett, The bone remodelling cycle, Annals of Clinical Biochemistry 55 (2018) 308-327.
